# 

**Published:** 1875-07-01

**Authors:** 


### The Chicago Medical Examiner.

### NOTICE TO SUBSCRIBERS.

### CONSOLIDATION.

The undersigned respectfully announce that they have become the publishers of "The Chicago Medical Examiner," heretofore published by N. S. Davis, M. D. and F. H. Davis, M. D., and that on and after August 1st, prox., it will be consolidated with

*The Chicago Medical Journal*, heretofore edited by J. Adams Allen, M. D., and Walter Hay, M. D.

These two periodicals will hereafter appear as

"The Chicago Medical Journal and Examiner,"

and be conducted by "The Chicago Medical Press Association," an incorporated Stock Company, composed of leading medical men of this city. It is proposed that we shall complete the subscriptions to The Medical Examiner for the present year by sending "The Chicago Medical Journal and Examiner" to all who have paid their subscriptions in full for 1875.

Subscribers to "The Medical Examiner" will, by the above arrangement, receive a journal of 80 pages monthly, instead of a 32 page journal twice a month, as heretofore.

If the above mentioned plan meets with your approval, please sign and return by early mail the inclosed Postal Card.

Yours Respectfully,

W. B. KEEN, COOKE & CO.,

*Publishers and Medical Booksellers*,

113 and 115 State Street,

CHICAGO.

*TERMS.— Yearly Subscriptions, Three Dollars, in advance. Single Number Fifteen Cents. All Communications should be addressed to Publishers* MEDICAL EXAMINER, 65 *Randolph Street, corner State, Chicago. Postage, Fifteen Cents per annum.*

Subscribe for your Journals through our office and obtain them at

**CLUB RATES.**

| | Regular Price. | With EXAMINER. |
|---|---|---|
| The London Lancet (Am. Rep.) | $5 00 | $7 00 |
| Philadelphia Medical and Surgical Reporter, (weekly) | 5 00 | 7 00 |
| " " Times (weekly) | 5 00 | 7 00 |
| New York Medical Record (weekly) | 5 00 | 7 00 |
| " " Journal (monthly) | 4 00 | 6 00 |
| Chicago Medical Journal (monthly) | 4 00 | 6 50 |
| " Journal of Nervous and Mental Disease (quarterly) | 4 00 | 6 00 |

Any other of our home or foreign Journals furnished with THE EXAMINER at ten per cent. discount from the gross subscription price. Address:

**PUBLISHERS MEDICAL EXAMINER,**
**65 East Randolph Street, CHICAGO.**

